# Impact of NAFLD on clinical outcomes in hepatocellular carcinoma treated with sorafenib: an international cohort study

**DOI:** 10.1177/17562848221100106

**Published:** 2022-09-30

**Authors:** Jessica Howell, Amit Samani, Binish Mannan, Saur Hajiev, Leila Motedayen Aval, Rebecca Abdelmalak, Vincent C. Tam, Dominik Bettinger, Robert Thimme, Tamar H. Taddei, David E. Kaplan, Max Seidensticker, Rohini Sharma

**Affiliations:** Department of Medicine, The University of Melbourne, St Vincent’s Hospital, Melbourne, VIC, Australia; Disease Elimination Program, Macfarlane-Burnet Institute, Melbourne, VIC, Australia; Department of Epidemiology and Preventive Medicine, Monash University, Melbourne, VIC, Australia; Department of Medical Oncology, Hammersmith Hospital, Imperial College Hospital NHS Trust, London, UK; Department of Surgery and Cancer, Imperial College London, London, UK; Department of Surgery and Cancer, Imperial College London, London, UK; Department of Surgery and Cancer, Imperial College London, London, UK; Department of Surgery and Cancer, Imperial College London, London, UK; Department of Oncology, Cumming School of Medicine, University of Calgary, Calgary, AB, Canada; University Medical Center Freiburg and Department of Medicine II, University of Freiburg, Freiburg, Germany; Berta-Ottenstein Programme, Faculty of Medicine, University of Freiburg, Freiburg, Germany; University Medical Center Freiburg and Department of Medicine II, University of Freiburg, Freiburg, Germany; Yale School of Medicine, New Haven, CT, USA; VA Connecticut Healthcare System, West Haven, CT, USA; Perelman School of Medicine, University of Pennsylvania and Corporal Michael J. Crescenz VA Medical Center, Philadelphia, PA, USA; Klinik und Poliklinik für Radiologie, Klinikum der Universität München, LMU München, Munchen, Germany; Consultant and Reader in Medical Oncology and Clinical Pharmacology, Department of Surgery and Cancer, Imperial College London, Hammersmith Campus, Du Cane Road, London W12 0NN, UK

**Keywords:** hepatocellular carcinoma, nonalcoholic fatty liver disease, sorafenib, survival, toxicity

## Abstract

**Background:**

The impact of nonalcoholic fatty liver disease (NAFLD) on overall survival (OS), treatment response and toxicity in patients with hepatocellular carcinoma (HCC) treated with sorafenib is unknown. We examined the impact of NAFLD on survival and toxicity in an international cohort of patients receiving sorafenib.

**Methods:**

Clinical and demographic data were collected from patients consecutively treated at specialist centres in Europe and North America. The impact of NAFLD on OS, sorafenib-specific survival and toxicity compared with other aetiologies of liver disease using multivariable Cox-proportional hazards and logistic regression modelling was assessed.

**Results:**

A total of 5201 patients received sorafenib; 183 (3.6%) had NAFLD-associated HCC. NAFLD-associated HCC patients were more likely to be older women (median age 65.8 *versus* 63.0 years, *p* < 0.01 and 10.4% *versus* 2.3%, < 0.01), with a median body mass index (BMI) of 29.4. After controlling for known prognostic factors, no difference in OS in patients with or without NAFLD was observed [hazard ratio (HR): 0.99, 95% confidence interval (CI): 0.84–1.18, *p* = 0.98]. NAFLD-associated patients had more advanced stage HCC when they commenced sorafenib [Barcelona Clinic Liver Class (BCLC) C/D 70.9% *versus* 58.9%, *p* < 0.01] and were more likely to be commenced on a lower starting dose of sorafenib (51.4 *versus* 36.4%, *p* < 0.01). There was no difference in sorafenib-specific survival between NAFLD and other aetiologies (HR: 0.96, 95% CI: 0.79–1.17, *p* = 0.96). Adverse events were similar between NAFLD and non-NAFLD HCC groups, including rates of greater than grade 2 hypertension (6.3% *versus* 5.8%, *p* = 1.00).

**Conclusion:**

Survival in HCC does not appear to be influenced by the presence of NAFLD. NAFLD-associated HCC derive similar clinical benefit from sorafenib compared with other aetiologies.

## Introduction

Hepatocellular carcinoma (HCC) is a major cause of cancer-related deaths worldwide and unlike other cancer types, incidence and mortality rates continue to rise.^1^ The vast majority of HCC deaths occur on a background of chronic liver disease (CLD).^2^ Currently, the most common causes of HCC worldwide are hepatitis B virus (HBV) infection, hepatitis C virus (HCV) infection and alcohol-related liver disease (ALD); however, nonalcoholic fatty liver disease (NAFLD)-related HCC incidence is rapidly rising in parallel with the global epidemic of obesity, type 2 diabetes and the metabolic syndrome and is poised to overtake HBV infection as the lead cause of HCC worldwide.^3–5^ In the United States, Europe and Australasia, NAFLD currently accounts for approximately 8–14% of HCC cases; although prevalence of NAFLD-related HCC is lower in Asian countries at present, a similar exponential trajectory is expected as prevalence of the metabolic syndrome increases, resulting in a dual burden of liver disease from NAFLD and viral hepatitis.^6,7^

NAFLD is characterised by excessive hepatic fat accumulation associated with insulin resistance in the absence of a secondary cause or significant alcohol consumption,^8^ defined by the presence of  >5% hepatocyte steatosis either histologically on liver biopsy, or imaging.^9^ Non-alcoholic steatohepatitis (NASH), part of the spectrum of nonalcoholic fatty liver disease (NAFLD), is characterised by steatosis with concomitant inflammation and hepatocyte ballooning and is associated with greater risk of adverse outcomes including cirrhosis and HCC.^8,9^ NAFLD is an independent risk factor for the development of HCC with an estimated incidence of 0.44 per 1000 persons/year, however the relative risk of HCC increases to 5.29 per 1000 persons/year for patients with evidence of steatohepatitis (NASH).^6^ Although a significant number of NAFLD-associated HCC occur in the absence of cirrhosis,^10,11^ when cirrhosis is present, the annual rate of HCC is estimated as high as 12.8%.^12–14^

Patients with NAFLD-associated HCC tend to be older than those with non-NAFLD HCC, have more extrahepatic comorbidities, but a lower prevalence of cirrhosis (only two-thirds of cases).^12,14^ The impact of the additional comorbidities associated with the metabolic syndrome on survival and response to standard HCC treatment are poorly defined.^13^ While 1 year mortality was higher in NAFLD compared with non-NAFLD HCC patients undergoing liver transplant in the SEER cohort,^15^ this was not the case with resection or ablation, where survival was similar. Not all studies investigating the impact of NAFLD on survival have accounted for the presence or absence of cirrhosis, itself a strong predictor of mortality in HCC.^16^ Moreover, response to specific treatment modalities has not been extensively explored.

Clinical relevance of disease aetiology to predict treatment response has already been demonstrated retrospectively for sorafenib, a multi-targeted tyrosine kinase inhibitor licenced for first-line treatment of HCC. Sub-group analysis of the pivotal Sorafenib Hepatocellular Carcinoma Assessment Randomized Protocol (SHARP) trial found that the hazard ratio (HR) for overall survival (OS) improvement for sorafenib *versus* placebo was better for those with HCV infection compared with HBV infection, while a recent meta-analysis of 3256 patients showed an improved OS for patients who were HCV positive and HBV negative.^17–19^ Neither analysis interrogated the clinical efficacy of sorafenib in patients with NAFLD-associated HCC. NAFLD is commonly associated with the metabolic syndrome, a cluster of hyperglycaemia/insulin resistance, obesity and dyslipidaemia, which carries an independent risk of cardiovascular mortality.^20–22^ Moreover, NAFLD is commonly associated with hypertension,^23^ a frequent side effect of sorafenib therapy,^17^ which may impede treatment adherence and target dosing in this population group. Finally, obesity and type 2 diabetes are independently associated with HCC,^24–28^ therefore, may impact prognosis and OS in patients with NAFLD-related HCC. The recently published IMbrave150 study^29^ demonstrates superiority of combination atezolizumab and bevacizumab over sorafenib for advanced HCC. However, subgroup analysis illustrates that nonviral patients did not benefit from immunotherapy combination, (HR: 0.71, 95% CI: 0.47–1.08), albeit small numbers and no information is given as to the proportion of NAFLD patients.^29^ Similarly, CheckMate049 did not show an advantage for nivolumab over sorafenib for the nonviral subgroup (HR: 0.95, 95% CI: 0.52–1.60).^30^ In addition, for many healthcare systems, the cost of the combination therapy will be prohibitive and sorafenib will remain in use particularly in countries in Asia where the burden of NAFLD is increasing.^7^ There is a need, therefore, to assess the therapeutic benefit of sorafenib in NAFLD-associated HCC.

We conducted a large, international multicentre cohort study to measure the impact of NAFLD on overall survival (OS) and sorafenib-specific survival in HCC compared with other aetiologies. We also assessed the incidence of sorafenib-related toxicities in patients with and without NAFLD.

## Materials and methods

In this multicentre cohort study, clinical and demographic data were collected from HCC patients who underwent treatment with sorafenib between 1 January 2007 and 31 December 2018 (*N* = 5201). Patients were consecutively recruited from three centres: Veterans Health Administration (VHA) Hospitals, USA (4903 patients, 90.0%);^31^ University Medical Centre Freiburg, Germany (183 patients, 3.4%)^32^ and Imperial College NHS Healthcare Trust, UK (132 patients, 2.4%). Data were also obtained from the recently published SORAMIC study (208 patients, 3.8%)^33^ and a consecutively recruited cohort of NAFLD-associated HCC patients from the Province of Alberta, Canada (18 patients, 0.3%).

Liver cirrhosis was defined by transient elastography using disease-specific liver stiffness measurement cutoffs or by liver biopsy histopathology.^34^ NAFLD was defined at each centre by either imaging findings or liver biopsy in accordance with EASL criteria^9^ in the absence of HCV or HBV infection or significant alcohol consumption.^35^ Where patients had more than one liver disease, patients with NAFLD and inactive or past liver disease of another aetiology that was no longer active at the time of HCC diagnosis were included in the NAFLD cohort; however, patients with NAFLD and other active liver disease were excluded from the study to reduce bias. Patients with more than one liver disease other than NAFLD were included in the non-NAFLD cohort.

Presence of diabetes mellitus was diagnosed by the managing clinicians at each site in accordance with the American Association of Clinical Endocrinology^36^ guidelines, defined by a blood glucose level ⩾200 mg/dl measured 2 h post 75 g oral glucose load; OR a random blood glucose level ⩾200 mg/dl plus symptoms of diabetes including polyurea, polydipsia or polyphagia, confirmed on a separate day of repeat testing.

Patients from Imperial College NHS Healthcare Trust were prospectively enrolled into a clinical database. Patient data from all other centres were collected following retrospective case review. No patients had received systemic therapy prior to sorafenib. Patients were excluded if they were lost to follow-up or if they were Child–Turcotte–Pugh (CTP) C stage cirrhosis at baseline. Patients in the SORAMIC study arm randomised to receive selective internal radiotherapy were also excluded. Patients were either started on sorafenib at a reduced dose, 400 mg once daily, or 400 mg twice daily (800 mg/day), depending on clinical assessment. The clinical trial protocol relating to SORAMIC has been previously published.^33^ Patients were censored at the time of death; no patients in the study received further systemic therapy after cessation of sorafenib during the study period.

Toxicity related to sorafenib, including hand–foot skin reaction (HFSR), diarrhoea, liver dysfunction, anorexia, fatigue and hypertension, was evaluated using the National Cancer Institute Common Terminology Criteria for Adverse Events 4.03 (CTCAE). Treatment with sorafenib continued until significant toxicity from treatment, disease progression or withdrawal of consent. Overall survival (OS) was taken from the date of HCC diagnosis to date of death or date of last follow-up. Sorafenib-specific survival was derived from the date of sorafenib commencement to date of death or date of last follow-up. The study protocol was approved by the institutional review board or ethics committee in each participating institution and was conducted in accordance with the Declaration of Helsinki (update 2004). The study protocol was approved by the Yorkshire & The Humber – Sheffield Research Ethics Committee in the United Kingdom (Reference 17/YH/0015), as well as the institutional review boards in each participating institution (Supplementary Information 1). The study was conducted in accordance with the Declaration of Helsinki (update 2004). Subjects provided informed consent in centres with prospective data collection, and in centres with retrospective data collection, consent was waived by the respective institutional review boards.

### Statistical analysis

Analysis was conducted in accordance with the STROBE criteria for observational studies (Supplementary Materials). The primary endpoints were OS and sorafenib-related survival and the main exposure was NAFLD-related HCC compared with HCC due to other aetiologies. Secondary outcome was the difference in incidence of sorafenib-related AEs between the NAFLD and non-NAFLD HCC groups. Given the small sample size of NAFLD patients, we performed a *post hoc* power calculation and determined with our sample size of 183 NAFLD and 5018 non-NAFLD patients, we had 80% power to detect a 20% difference in HR for overall survival between the NAFLD and non-NAFLD groups. OS was defined from the date of HCC diagnosis to death from any cause. Sorafenib-related survival was defined as the time from commencement of sorafenib to date of death from any cause. There were no patients lost to follow-up within the study time period.

Potential confounding variables were also collected, including demographic (site, age, gender), clinical [including aetiology of liver disease, liver disease severity (Child–Pugh class), presence of cirrhosis, Barcelona Clinic Liver Class (BCLC) stage HCC], comorbidities [body mass index (BMI), diabetes, cardiovascular disease, hypertension] and treatment-related factors (previous treatment HCC and modality, response to treatment, adverse effects). Non-parametric continuous variables were expressed as median (interquartile range, IQR). Categorical variables were expressed as frequencies with percentages. Variables were compared between NAFLD and other aetiologies using the nonparametric Mann–Whitney U test for continuous variables and *χ*^2^-test or Fisher’s exact test for categorical variables, as appropriate. A crude (or unadjusted) HR for the impact of NAFLD on OS and sorafenib-related survival was determined using log-rank test. Bivariable stratification was used to determine potential confounders and interaction between other prognostic variables, NAFLD status and OS and sorafenib-related survival. A multivariable Cox proportional hazards model was then built to adjust for potential confounding factors identified on bivariable analysis and *a priori* from the literature, including BCLC stage (AB *versus* CD), serum α-fetoprotein (AFP; ⩽ or  >400 ng/dl),^37^ tumour size (⩽ or  >7 cm) and presence of cirrhosis.^16^ The univariable survival function by aetiology and potential prognostic factors was plotted using Kaplan–Meier curves and the Log-rank test, followed by stepwise backward multivariable Cox regression modelling. All *p*-values were derived from two-tailed tests, and *p* < 0.05 was defined as statistically significant. All statistical analysis was conducted using SPSS statistical package version 26 (SPSS Inc., Chicago, IL, USA).

## Results

### Baseline characteristics

A total of 5201 patients with HCC were included in the study; 183 patients (3.4%) had NAFLD-associated HCC. The mean age of the study population was 63.1 years (range 20–92 years). The baseline characteristics of the study cohort stratified according to NAFLD are presented in Table 1. The proportion of HCC patients receiving sorafenib with NAFLD varied by site; all HCC cases from the Canadian cohort had NAFLD HCC (*n* = 18), compared with 2% of the VA cohort (74 of 4688 HCC cases). The commonest cause of underlying liver disease was HCV infection (*n* = 2640, 50.8%) followed by alcohol-related liver disease (*n* = 2213, 42.5%) and HBV infection (*n* = 273, 5.2%). Fifty (27%) NAFLD HCC patients had a history of inactive liver disease aetiology: 38 (21%) had previous alcohol-related liver disease, 20 (11%) had past HCV infection and 5 (3%) had inactive or past hepatitis B infection. Patients with NAFLD-associated HCC were more likely to be female (10.4% *versus* 2.3%, *p* < 0.01) and diagnosed at an older age (65.8 *versus* 63.0 years, *p* < 0.01). The majority of HCC patients had cirrhosis (97%); however, those with NAFLD had a lower proportion of cirrhosis (85.3%) compared with those without NAFLD (97%, *p* < 0.01). The distribution of diabetes and cardiovascular disease was similar between the NAFLD and non-NAFLD cohorts (diabetes: 47.1 *versus* 43.6%, *p* = 0.3 and cardiovascular disease: 10.8 *versus* 9.9%, *p* = 0.79).

**Table 1.** Baseline characteristics of the study population at time of initiation of sorafenib.

Baseline characteristic	All patients (%), range *N* = 5201	NAFLD (%) *N* = 183 (3.6)	Other (%) *N* = 5018 (96.4)	*p*-value
Centre				<0.01
Unites States	4688 (90.2)	74 (40.4)	4614 (92.0)	
SORAMIC cohort	207 (4.0)	29 (15.8)	178 (3.5)	
Germany	180 (3.5)	34 (18.6)	146 (2.9)	
United Kingdom	107 (2.1)	28 (15.3)	79 (1.6)	
Canada	18 (0.3)	18 (9.8)	–	
Age, years, median (IQR)	63.1 (9)	65.8 (15.1)	63.0 (9)	<0.01
Sex				<0.01
Male	5070 (97.5)	164 (89.6)	4906 (97.7)	
Female	132 (2.5)	19 (10.4)	113 (2.3)	
BMI, median (IQR)	26.4 (7.03)	26.9 (6.8)	26.4 (7.1)	0.1
Diabetes (*N* = 4729)				0.3
Absent	2665 (51.2)	55 (52.9)	2610 (56.4)	
Present	2064 (39.7)	49 (47.1)	2015 (43.6)	
Hypertension (*N* = 4560)				<0.01
Absent	134 (2.6)	11 (12.2)	123 (2.7)	
Present	4516 (86.8)	79 (87.8)	4437 (97.3)	
Cardiovascular disease^a^ (*N* = 4688)				0.79
Absent	4224 (90.1)	66 (89.2)	4158 (90.1)	
Present	464 (9.9)	8 (10.8)	456 (9.9)	
Child Turcotte Pugh Class (*N* = 5183)				0.19
A	3287 (63.4)	113 (68.5)	3174 (63.3)	
B	1895 (36.6)	52 (31.5)	1843 (36.7)	
Barcelona Clinic Liver Cancer (*N* = 4769)				
A	258 (5.4)	10 (5.6)	248 (5.4)	<0.01
B	1677 (35.2)	42 (23.6)	1635 (35.6)	
C	2690 (56.4)	109 (61.2)	2581 (56.2)	
D	143 (3.0)	17 (9.6)	126 (2.7)	
Maximum tumour diameter (*N* = 3093)				0.79
⩽ 7 cm	2027 (65.6)	105 (66.9)	1922 (65.5)	
> 7 cm	1065 (34.4)	52 (33.1)	1031 (34.5)	
Portal vein thrombus (*N* = 4584)				0.72
Absent	3216 (70.2)	108 (68.8)	3108 (70.2)	
Present	1367 (29.8)	49 (31.2)	1318 (29.8)	
AFP (μg/dl) (*N* = 4806)				0.62
⩽ 400	3034 (63.1)	103 (65.2)	2931 (63.1)	
> 400	1771 (36.9)	55 (35.8)	1716 (36.9)	
Cirrhosis (*N* = 5198)				
Absent	179 (3.4)	27 (14.8)	151 (3.0)	<0.01
Present	5019 (96.6)	156 (85.3)	4863 (97.0)	
Metastases (*N* = 5172)				
Absent	4150 (80.3)	128 (73.1)	4022 (80.5)	0.02
Present	1021 (19.7)	47 (26.9)	974 (19.5)	
Previous Treatment				
Resection^a^	55 (15.3)	13 (15.3)	42 (16.9)	0.86
Radiofrequency ablation	296 (5.9)	17 (10.8)	279 (5.8)	<0.01
Transarterial chemoembolisation	1398 (27.7)	69 (42.3)	1329 (27.2)	<0.01
Y90	5 (0.1)	0 (0.0)	5 (0.1)	1.0
Sorafenib dose				<0.01
800 mg	3278 (63.0)	89 (48.6)	3189 (63.6)	
400 mg	1922 (37.0)	94 (51.4)	1828 (36.4)	
Mean duration of sorafenib treatment (months)	5.3	6.5	5.3	0.03

AFP, α-fetoprotein; BMI, body mass index; IQR, interquartile range; NAFLD, nonalcoholic fatty liver disease.

a Cardiovascular disease data only available for the Veterans Affairs cohort.

### Impact of NAFLD on survival outcome

At the time of commencing sorafenib, the NAFLD-associated HCC population had more advanced stage disease both in terms of BCLC C/D stage (70.8 *versus* 58.9%, *p* < 0.01) and the presence of distant metastases compared with other aetiologies (26.9 *versus* 19.5%, *p* = 0.02). The NAFLD-associated HCC patients were also more likely to have received previous therapy with RFA and TACE (53.1 *versus* 33.0%, *p* < 0.01; Table 1).

**Figure 1.**
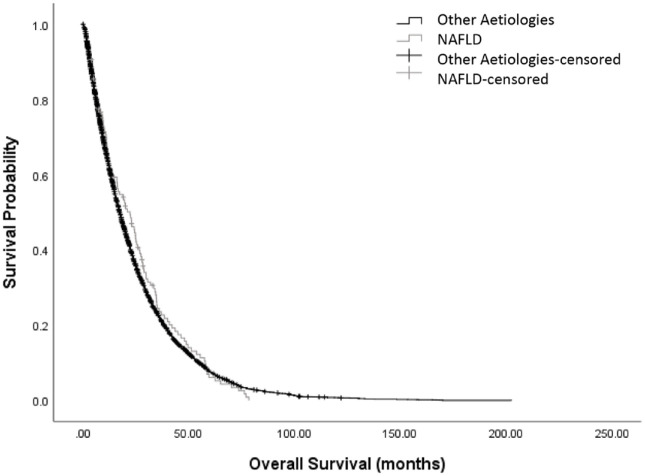
Kaplan–Meier curves illustrating the prognostic relationship of presence of NAFLD and other aetiologies with overall survival in patients with HCC.

At the time of analysis, all patients within the cohort had died. The median OS was 17.6 months (95% CI: 16.9–18.3). No significant difference was observed between the OS of NAFLD and non-NAFLD HCC patients: median OS was 19.3 months (95% CI: 14.7–23.9) in the NAFLD cohort compared with 17.6 months (95% CI: 16.9–18.3) in those without NAFLD (unadjusted HR: 0.99, 95% CI: 0.84–1.18, *p* = 0.98; Figure 1).

**Figure 2.**
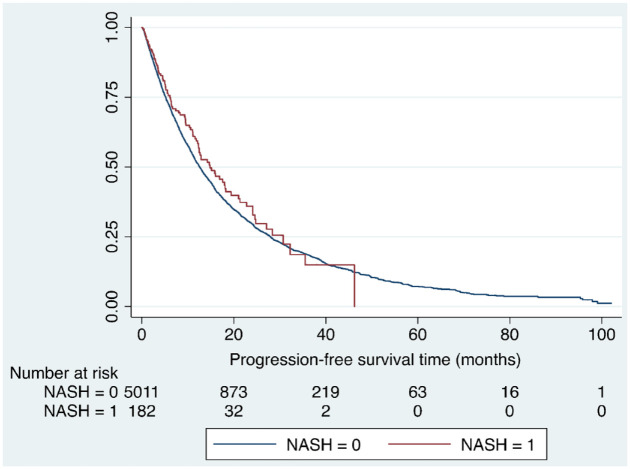
Kaplan–Meier curve illustrating difference in progression-free survival in people with NAFLD compared with people without NAFLD on sorafenib therapy (HR: 0.89, 95% CI: 0.72–1.11, *p* = 0.24).

A total 3278 patients (63.0%) were started on sorafenib 800 mg/day and 1922 (37.0%) received a reduced dose of 400 mg/day, with a median follow-up time of 15.2 months. Patients with NAFLD-associated HCC were more likely to be started on a reduced dose of sorafenib than non-NAFLD HCC patients (51.4% *versus* 36.4%, *p* < 0.001). The median duration of sorafenib treatment was 5.2 months (range 0.1–83.6 months), with patients with NAFLD being on treatment for significantly longer compared with other aetiologies (6.48 *versus* 5.31 months, *p* = 0.033). However, progression-free survival did not differ between the NAFLD and non-NAFLD groups; median progression-free survival was 14.8 months (6.1, 30.7 months) in the NAFLD group compared with 12.6 months (5.2, 27.9 months) in the non-NAFLD group (*p* = 0.24; Figure 2).

Median sorafenib-specific survival was 7.69 months (95% CI: 0.79–8.01). On univariable analysis factors that were significantly associated with worse sorafenib survival were presence of cirrhosis (HR: 1.27 95% CI: 1.08–1.50, *p* = 0.05), presence of extrahepatic metastases (HR: 1.41, 95% CI: 1.31–1.51, *p* < 0.001), CTP B stage cirrhosis (HR: 1.13, 95% CI: 1.06–1.19, *p* < 0.001) and BCLC C/D stage HCC (HR: 1.49, 95% CI: 1.41–1.59, *p* < 0.001). Factors associated with improved sorafenib survival were dose of sorafenib (HR: 0.92, 95% CI: 0.86–0.97, *p* = 0.03), duration of sorafenib therapy (HR: 0.92, 95% CI: 0.91–0.92, *p* < 0.001) and previous therapy with resection, radiofrequency ablation or transarterial chemoembolisation (HR: 0.81, 95% CI: 0.76–0.86, *p* < 0.001; Table 2). Presence of diabetes and cardiovascular disease were not associated with sorafenib-specific survival. The final Cox proportional hazards multivariable model showed no significant impact of NAFLD status on survival in patients receiving sorafenib (HR: 0.96, 95% CI: 0.79–1.17, *p* = 0.69) when adjusted for BCLC and CTP stage, tumour size  >7 cm, AFP > 400 IU/l, presence of cirrhosis, presence of extrahepatic metastases, dose, duration of therapy or receipt of previous therapy (Table 3).

**Table 2.** Clinical variables associated with sorafenib-related survival on univariate analysis in patients with advanced stage HCC.

Predictor	Median overall survival (months)	Univariable models	
		Hazard ratio (95% CI)	*p*
NAFLD *versus* other aetiologies	9.51 *versus* 7.66	0.99 (0.84–1.18)	0.98
Age (<75 *versus* ⩾75years)	7.79 *versus* 7.14	1.06 (0.97–1.16)	0.2
BCLC stage C or D *versus* 0, A or B	5.95 *versus* 10.13	1.49 (1.41–1.59)	<0.001
CTP class (B *versus* A)	4.51 *versus* 10.07	1.13 (1.06–1.19)	<0.001
Tumour size ⩾7 *versus* <7 cm (*N* = 3091)	5.36 *versus* 9.51	1.00 (0.93–1.09)	0.92
AFP ⩾400 *versus* <400 IU/dl (*n* = 4804)	5.76 *versus* 9.87	1.06 (1.00–1.13)	0.052
Cirrhosis (present/absent)	7.59 *versus* 11.41	1.27 (1.08–1.50)	0.005
Metastases (present/absent)	5.36 *versus* 8.26	1.41 (1.31–1.51)	<0.001
PVT (present; *N* = 4582)	7.89 *versus* 7.43	1.00 (0.94–1.07)	0.56
Diabetes (present/absent)	7.70 *versus* 7.73	0.99 (0.93–1.04)	0.63
Hypertension (present/absent)	12.2 *versus* 13.9	1.04 (0.87–1.25)	0.65
Cardiovascular disease^a^ (present/absent)	6.74 *versus* 7.63	1.05 (0.95–1.16)	0.35
Previous treatment (yes/no)	9.67 *versus* 6.61	0.81 (0.76–0.86)	<0.001
Starting dose (800 mg *versus* 400 mg)	8.09 *versus* 7.00	0.92 (0.86–0.97)	0.003
Dose reduction (yes/no)	5.90 *versus* 8.93	0.92 (0.91–0.92)	<0.001
Treatment response^b^ (yes/no)	8.00 *versus* 7.70	0.91 (0.39–2.11)	0.819
Duration of treatment	N/A	0.92 (0.91–0.92)	<0.001

AFP, α-fetoprotein; BCLC, Barcelona Clinic Liver Class; CI, confidence interval; CTP: Child–Turcotte–Pugh; HCC, hepatocellular carcinoma; NAFLD, nonalcoholic fatty liver disease; PVT: portal vein thrombus.

a The impact of cardiovascular disease on sorafenib-specific survival was only assessed in the Veterans Affairs cohort.

b Treatment response was defined as complete response, partial response or stable disease per RECIST criteria on imaging [CT quad phase or contrast-enhanced magnetic resonance imaging (MRI) of liver].

**Table 3.** Multivariable predictors of sorafenib-related survival when controlling for NAFLD status.

Predictor	Crude HR (95% CI)	*p*-value
NAFLD	0.99 (0.85–1.16)	0.92
	Adjusted HR (95% CI)	
NAFLD	0.96 (0.79–1.17)	0.69
BCLC stage C or D *versus* 0, A or B	1.69 (1.58–1.80)	<0.001
CTP class (B *versus* A)	1.77 (1.65–1.89)	<0.001
Cirrhosis (presence/absence)	1.49 (1.09–2.04)	0.013
Previous treatment (Y/N)	0.89 (0.82–0.95)	0.001
Dose (800 mg *versus* 400 mg)	0.93 (0.86–0.99)	0.024
Duration of treatment	0.91 (0.90–0.91)	<0.001

BCLC, Barcelona Clinic Liver Class; CI, confidence interval; CTP: Child–Turcotte–Pugh; HR, hazard ratio; NAFLD, nonalcoholic fatty liver disease.

Information on history of acute myocardial infarction and congestive cardiac failure was available for the VHA cohort (*N* = 4903). In this cohort, 408 (8.3%) had heart failure and 97 (2.0%) had a history of myocardial infarction. When considering the VHA cohort only, the presence of cardiac events had no impact on either overall OS (HR: 1.04, 95% CI: 0.94–1.15, *p* = 0.44) or sorafenib-specific survival (HR: 1.04, 95% CI: 0.94–1.15, *p* = 0.44). There was no difference in the incidence of cardiac events between the NAFLD and other aetiologies in the VHA cohort (10.8% *versus* 9.9%, *p* = 0.69).

### Sorafenib-related side effects

Overall, progressive disease was the main reason for sorafenib cessation (43.7%) followed by toxicity (20.0%) and this was also the case in the sub-population with NAFLD-associated HCC (cessation due to progressive disease 52% and toxicity 21.3%), with no significant difference between the two cohorts. Of interest, a small but significant group of patients within the NAFLD cohort stopped sorafenib due to ‘patient preference’ when compared with the non-NAFLD cohort [10 (6.7%) *versus* 41 (0.9%), *p* < 0.001). Sorafenib was well tolerated; the most common severe (Grade ⩾2) AEs experienced were diarrhoea and fatigue, occurring in 26.3% and 14.7% of all patients, respectively. Regarding specific AEs, the NAFLD group had a significantly lower incidence of HFSR (3.8% *versus* 12.4%, *p* = 0.032) compared with other aetiologies (Table 4). Of interest, there was no difference in the incidence of grade 2–5 hypertension between the two groups (6.3 *versus* 5.8%, *p* = 0.10).

**Table 4.** Incidence of adverse events to sorafenib (NCI-CTC AE version 4.03).

	AEs in NAFLD, *n* (%)		AEs in other causes, *n* (%)		*p*-value
	Grade <2	Grade ⩾2	Grade <2	Grade ⩾2	
HFSR	77 (96.3)	3 (3.8)	197 (87.6)	28 (12.4)	**0.03**
Rash	43 (100.0)	0 (0.0)	130 (98.5)	2 (1.5)	1.00
Mucositis	79 (98.8)	1 (1.3)	223 (99.1)	2 (0.9)	1.00
Hypertension	75 (93.8)	5 (6.3)	212 (94.2)	13 (5.8)	1.00
Anorexia	60 (96.8)	2 (3.2)	224 (99.6)	1 (0.4)	0.12
Fatigue	69 (83.3)	11 (13.8)	191 (84.9)	34 (15.1)	0.86
Diarrhoea	63 (78.8)	17 (21.3)	160 (71.1)	65 (28.9)	0.24
Constipation	62 (100.0)		225 (100.0)		
Liver dysfunction	35 (100.0)	0 (0.0)	115 (99.1)	1 (0.9)	1.00
Other	39 (97.5)	1 (2.5)	113 (97.4)	3 (2.6)	1.00

HFSR, hand–foot skin reaction; NAFLD, nonalcoholic fatty liver disease; NCI-CTC AE, National Cancer Institute Common Terminology Criteria for Adverse Events.

## Discussion

The metabolic syndrome is a growing epidemic worldwide and is not only associated with increasing risk of cardiovascular disease and endocrine disorders but also of NAFLD and gastrointestinal malignancies, in particular HCC.^38,39^ Despite the increasing numbers of patients with NAFLD-associated HCC, there is a paucity of data in the literature regarding clinical outcomes with treatment. The SEER cohort considered outcomes in NAFLD–HCC patients undergoing transplantation, ablation and surgery.^15^ However, as highlighted in this study and by others, patients with NAFLD-associated HCC often present with advanced stage disease and at older age and are, therefore, only suitable for systemic therapy.^11^ The use of systemic therapy in this cohort is of particular consideration as patients with NAFLD-associated HCC patients have associated cardiovascular comorbidities that may impact on treatment outcome.^11^ To investigate the impact of NAFLD on both OS and sorafenib-specific survival, we conducted a large, multinational study comparing outcomes for NAFLD and other aetiologies, adjusting for known prognostic factors in HCC.

Consistent with previous studies, we report that patients with NAFLD-associated HCC were more likely to be female and older at the time of HCC diagnosis, and were less likely to have underlying cirrhosis.^40–42^ At the time of commencement of sorafenib, NAFLD patients had more advanced stage disease both in terms of BCLC and the presence of metastases. NAFLD patients had also undergone more interventional therapies, RFA and TACE, prior to commencing sorafenib. Several studies have reported that NAFLD patients present with HCC at a more advanced stage.^13,43,44^ As these patients can develop HCC in the absence of cirrhosis and these patients do not currently fulfil criteria for HCC surveillance programmes, this may contribute to the higher proportion of NAFLD patients presenting with advanced stage HCC.^11,45^ This concept is supported by a retrospective study that compared cirrhotic and noncirrhotic NAFLD patients, and observed larger tumour sizes in those who were noncirrhotic.^45^ In addition, we observed that NAFLD patients were more likely to undergo RFA and TACE prior to receiving sorafenib, and that was an independent prognostic factor suggesting that NAFLD–HCC is not intrinsically more malignant and suggests a longer duration of malignant disease. It is also likely that patients that have undergone multiple therapies will have preserved underlying liver function, with CTP class shown to be an independent prognostic factor.

We observed no difference in OS or sorafenib-related survival between NAFLD-associated HCC and HCC from other aetiologies, after adjusting for known adverse prognostic factors. This is consistent with the studies by Piscaglia and Tokushige who compared survival in NAFLD–HCC with HCV–HCC and observed no difference in OS following propensity score matching.^11,46^ These findings are consistent with a study of TACE where underlying NAFLD did not impact on survival or toxicity;^47^ however, this has not previously been explored in HCC patients receiving systemic therapy. It is well described that patients with the metabolic syndrome and NAFLD have high rates of cardiovascular morbidity and mortality compared with those without NAFLD,^48^ and NAFLD patients with HCC have high rates of comorbid cardiovascular disease.^43^ While we only had cardiovascular outcome data for the Veterans Affairs cohort, it is reassuring that there did not appear to be an increased risk of cardiovascular-related death in the patients with NAFLD receiving sorafenib. The sorafenib-specific survival in our study is lower compared with the SHARP and Asia Pacific trial publications.^17,49^ This is likely to be due to the inclusion of a significant number of patients with CTP B liver dysfunction (36.6%), and our data are in line with a meta-analysis by McNamara and colleagues^50^ who investigated the benefit of sorafenib across all CTP status groups and reported a similar OS of 7.2 months.

NAFLD patients were treated for a significantly longer duration after being commenced on a lower starting dose, and while information regarding subsequent dose changes or cumulative dose was not available, a recent study by Tovoli and colleagues^51^ illustrates that tailoring dose to the individual patient results in a longer duration of therapy and improved OS supporting our finding that sorafenib duration was an independent prognostic factor. Given the significantly lower starting dose of sorafenib in the NAFLD patients, one may speculate that the treating physicians were cautious about adverse outcomes in NAFLD and altered their prescribing accordingly. This may also be reflected by the significant number of NAFLD patients that ceased sorafenib due to ‘patient preference’. No further details were recorded by centres and while the numbers are small, it could be inferred that these patients were more likely to stop therapy due to other comorbidities, a concept that requires further exploration in prospective studies.

A key consideration with any systemic therapy is the incidence of adverse events, particularly as the primary goal of therapy remains that of palliation. One of the commonest side effects of sorafenib is hypertension^17^ and we anticipated a higher incidence of this toxicity in the NAFLD cohort who were likely to have baseline hypertension due to the metabolic syndrome; however, this did not transpire with the incidence of hypertension being the same in both patient groups. The only difference in toxicity was observed with HSFR, for which we noted a significantly reduced incidence of moderate–severe rates of toxicity in the NAFLD patients. The effect of dose on the development of HFSR is not well delineated, but based on clinical experience, dose reduction and discontinuation of sorafenib reduces the severity of HFSR. Patients with NAFLD-associated HCC were more likely to be commenced on a lower dose of sorafenib and it is possible that this translated to a lower incidence of toxicity observed than was expected. Other medication usage was not routinely recorded in this study; therefore, it is possible that patients with NAFLD and underlying hypertension were already on antihypertensives when they commenced sorafenib, which may have confounded our ability to detect sorafenib-mediated hypertension. Given the small numbers of patients investigated in the NAFLD cohort, this finding is of interest yet remains to be validated in larger studies.

A key limitation of this study and of any study in NAFLD pertains to the definition of NAFLD used. Previous guidelines have defined NAFLD as the presence of steatosis in >5% of hepatocytes in the absence of significant ongoing or recent alcohol consumption and other known causes of liver disease,^9^ a definition that relies on exclusion of other pathologic factors. However, it is increasingly recognised that NAFLD may coexist with other liver diseases such as hepatitis B.^52^ A recent consensus guideline has suggested alternate diagnostic criteria that aim to reduce subjectivity in diagnosis and are based on evidence of hepatic steatosis, in addition to one of the following three criteria, namely overweight/obesity, presence of type 2 diabetes mellitus or evidence of metabolic dysregulation.^53^ In this study, NAFLD status was defined by individual centres using EASL guidelines;^9^ patients with mixed pathology or cryptogenic fibrosis were excluded from the NAFLD cohort to reduce the potential for misclassification of NAFLD patients or potential confounding effects of other underlying aetiologies when assessing the impact of NAFLD status on survival in patients receiving sorafenib. Although no patients with NAFLD according to EASL criteria^9^ were included in the non-NAFLD HCC cohort, it is possible some of the non-NAFLD patients may have had hepatitis steatosis due to type 3 HCV infection or alcohol-related liver disease and the impact of this on survival in HCC patients, in the absence of other diagnostic features of NAFLD, remains uncertain.

There are several limitations to our study. Data collected for the Veterans Affairs cohort that comprised the greatest proportion of study patients were retrospective, which may potentially lead to under-reporting of the adverse effects of treatment. The cohorts included in the study were all from specialist HCC referral centres; therefore, selection bias towards more advanced stage liver disease and HCC must be considered. In particular, within the NAFLD cohort possible referral centre bias (more cirrhosis cases recruited from tertiary liver centres) and surveillance bias (recognition of need for surveillance in cirrhotics but no indication at present for screening NAFLD patients without cirrhosis) may have confounded the apparent impact of NAFLD status on survival in HCC patients. Although patients were consecutively recruited at all centres, there is the potential for selection bias and unmeasured confounding. Approaches such as propensity score analysis can be very useful to reduce the impact of unmeasured confounding in observational studies and would be a recommended approach for future, large registry-based studies with a greater sample size of NAFLD patients. Finally, although the overall cohort was large, the number with NAFLD was relatively low that may have limited our power to detect a significant difference in survival between NAFLD and non-NAFLD patients. Our *post hoc* power calculation determined we could detect a 20% or greater difference in HR for overall survival between the NAFLD and non-NAFLD groups; therefore, smaller effect sizes may have not reached statistical significance (type II error).

However, the key strengths of this paper are the global nature of this study and the large patient cohort treated with sorafenib allowing us to explore survival in this increasingly large group of patients who due to their comorbidities may be less suitable for surgical therapy. Our study was also broad in its criteria for enrolment and is, therefore, a fair reflection of clinical practice. Patients were also enrolled consecutively in all centres to reduce selection bias. Since all centres were tertiary referral centres, the study benefitted from specialist physician expertise. While we acknowledge that newer combination immunotherapy treatments have superseded sorafenib as first line in many countries following the IMBrave 150 trial data,^29^ the role of sorafenib in the management of advanced stage HCC is still very relevant. The presence of varices secondary to portal hypertension precludes the use of combination atezoluzimab/bevacizumab.^29^ Moreover, there is increasing recognition that NAFLD-associated HCC may not respond to immunotherapy, a concept that is supported by preclinical evidence that NAFLD is associated with a more immunosuppressive microenvironment with a selective loss of CD4^+^ and effector memory cells, and an increase in CD8^+^ T lymphocytes as a result of dysregulated lipid metabolism that results in a impaired response to immunotherapy.^54,55^ Current guidelines now position sorafenib as second- or third-line therapy following first-line immunotherapy failure.^56^ Moreover, in middle-income countries where sorafenib is the only systemic agent available for HCC treatment, these data are particularly relevant as we witness a rapid increase in disease burden from NAFLD and associated HCC.^6,7^

The impact of the underlying aetiology of liver disease on OS is of key importance in determining patient prognosis. Patients with HCC have two competing pathologies of prognosis – the cancer itself and the underlying liver disease. From our study it is clear that NAFLD patients receive similar benefit from sorafenib and are not at a greater risk of death. In future studies, it would be of significant interest to record the cause of death in this patient group to ascertain whether mortality is limited by cancer or other comorbidities.

## Conclusion

This large international cohort study shows that patients with NAFLD-associated HCC are more likely to be older, have larger tumours and present at a more advanced stage. Nonetheless, these patients have an equivalent OS benefit with sorafenib compared with other underlying aetiologies, with acceptable overall rates of toxicity. These data reiterate that patients with NAFLD-associated HCC derive similar clinical benefit with sorafenib without the need for dose reduction.

## Supplemental Material

sj-docx-1-tag-10.1177_17562848221100106 – Supplemental material for Impact of NAFLD on clinical outcomes in hepatocellular carcinoma treated with sorafenib: an international cohort study Supplemental material, sj-docx-1-tag-10.1177_17562848221100106 for Impact of NAFLD on clinical outcomes in hepatocellular carcinoma treated with sorafenib: an international cohort study by Jessica Howell, Amit Samani, Binish Mannan, Saur Hajiev, Leila Motedayen Aval, Rebecca Abdelmalak, Vincent C. Tam, Dominik Bettinger, Robert Thimme, Tamar H. Taddei, David E. Kaplan, Max Seidensticker and Rohini Sharma in Therapeutic Advances in Gastroenterology

## Declarations

**Ethics approval and consent to participate:** Not applicable.

**Consent for publication:** Not applicable.

**Availability of data and materials:** Not applicable.
