# Supplementary material for: Impact of NAFLD on clinical outcomes in hepatocellular carcinoma treated with sorafenib: an international cohort study
Source: Ther Adv Gastroenterol. 2022 Sep 30;15:17562848221100106. doi: 10.1177/17562848221100106 (PMC9527996; doi:10.1177/17562848221100106)
Supplement: sj-docx-1-tag-10.1177_17562848221100106 – Supplemental material for Impact of NAFLD on clinical outcomes in hepatocellular carcinoma treated with sorafenib: an international cohort study [file sj-docx-1-tag-10.1177_17562848221100106.docx]

**Supplementary Information 1:** Ethics committees in participating institutions that approved the study.

- Local Ethics Committee of the University Hospital of Freiburg, Germany.
- Institutional Review Board at the VA Connecticut Healthcare System (West Haven, CT) and the Corporal Michael J. Crescenz VA Medical Center (Philadelphia, PA).
- IRCCS Istituto Clinico Humanitas Local Ethical Committee, Milan, Italy
- Institutional Review Board of National Cancer Centre, Goyang, Korea
- Ethics Committee of the University Hospital Maggiore of Charity of Novara, Italy
- Kindai University Hospital Institutional Review Board, Japan
